# Hydrogen Attenuates Myocardial Injury in Rats by Regulating Oxidative Stress and NLRP3 Inflammasome Mediated Pyroptosis

**DOI:** 10.7150/ijms.61329

**Published:** 2021-07-25

**Authors:** Hongxiao Yang, Shuang Liu, Huijun Du, Zi Hong, Yajing Lv, Chaoqun Nie, Wei Yang, Yunan Gao

**Affiliations:** 1 Department of Cardiology, the Fourth Affiliated Hospital of Harbin Medical University, Harbin, Heilongjiang, China; 2 Department of Cardiology, the First Affiliated Hospital of Harbin Medical University, Harbin, Heilongjiang, China

**Keywords:** hydrogen, acute myocardial infarction, oxidative stress, pyroptosis

## Abstract

**Purpose:** Hydrogen (H_2_) is an antioxidant with anti-inflammatory and apoptosis functions.This study aimed to estimate the effects of H_2_ on acute myocardial infarction (AMI) in rats and its association with the inhibition of oxidative stress and cardiomyocyte pyroptosis.

**Methods:** Sixty-four rats were randomly divided into three groups (Sham, AMI, and H_2_). The left anterior descending coronary artery (LAD) of rats in the AMI and H_2_ groups was ligated, while rats in the Sham group were threaded without ligation. In addition, 2% H_2_ was administered by inhalation for 24 h after ligation in the H_2_ group. Transthoracic echocardiography was performed after H_2_ inhalation, followed by collection of the serum and cardiac tissue of all rats.

**Results:** H_2_ inhalation ameliorated the cardiac dysfunction, infarct size and inflammatory cell infiltration caused by AMI. Meanwhile, H_2_ inhalation reduced the concentration of serum Troponin I (TnI), brain natriuretic peptide (BNP), reactive oxygen species (ROS), cardiac malondialdehyde (MDA), and 8-OHdG. In addition, H_2_ inhalation inhibited cardiac inflammation and pyroptosis relative proteins expression.

**Conclusion:** H_2_ effectively promoted heart functions in AMI rats by regulating oxidative stress and pyroptosis.

## Introduction

Acute myocardial infarction (AMI) remains a leading cause of heart failure (HF) and cardiac death. A reperfusion treatment strategy for AMI can minimize irreversible cardiac injury, reduce the infarct size, and improve heart prognosis; however, the risk of HF remains. 1 Even after successful recanalization, ischemic regions of the myocardium remain in nearly two-thirds of patients because of microvascular obstruction (MVO), which is closely related to a larger infarct size, adverse ventricular remodeling, and worse clinical outcomes. 2-4 In addition, most patients missed the best chance of treatment, which enhances the symptoms and can lead to death. Therefore, the prevention and treatment of continuous myocardial ischemia should be strictly controlled. 5

AMI adversely affects cardiomyocytes and heart function by ischemia and ischemia/reperfusion (I/R) injury. Irreversible ischemic stress can lead to progressive myocardial damage and increase membrane permeability. 6-7 Residual oxygen in the ischemic area of the myocardium is depleted in seconds in the absence of blood supply during AMI. Additionally, mitochondria in the ischemic area are no longer able to produce ATP without oxygen, leading to calcium overload and subsequent oxidative stress. 8 Inflammation, which is closely related to reactive oxygen species (ROS) production, plays an important role in ventricular remodeling. 9,10 Activation of the inflammasome and infiltration of leukocytes are observed in the marginal area where AMI occurred, 11 indicating that preventing the inflammatory response in the myocardial ischemic area might be a therapeutic strategy for AMI.

Pyroptosis is a caspase1-dependent cell death, characterized by cell membrane pores mediated by GSDMD-N, DNA breaks, and the release of pro-inflammatory cytokines. 12 Pyroptosis plays a crucial role in the pathogenesis of many cardiovascular diseases, including AMI, hypertension, cardiomyopathy, and HF. 13-16 Recently, an autopsy of the human heart showed that inflammatory bodies are activated when AMI occurs, with apoptosis-associated speck-like protein containing a cARd (ASC) aggregation. 11 Acute cardiomyocyte necrosis of an infarcted heart produces damage-associated molecular patterns, triggering a strong inflammatory response that is mediated through inflammasomes. 17

The inflammasome is a complex composed of multiple proteins that plays an important role in inflammatory response. Nod-like receptor (NLR) family pyrin domain containing protein 3 (NLRP3), one of the most widely studied inflammasomes; it produces a strong inflammatory response to diverse danger signals that are triggered by exogenous and endogenous pathogens, ROS, and other inflammatory substances. 13 Activated NLRP3 recruits the adaptor protein ASC and procaspase-1 to form a multi-protein complex, which leads to the activaton of caspase-1. Activated caspase-1 cleaves Gasdermin D into a peptide containing the N-terminal active domain, induces cell membrane perforation, cell rupture, and the contents release, causes inflammation. Additionally, it cleaves the pro-forms of interleukin (IL)-1β and IL-18 into their mature forms and recruits inflammatory cells to aggregate and expand the inflammatory response. NLRP3 has been associated with left ventricular remodeling after AMI; however, while its inhibitors and downstream effectors (IL-1β and IL-18) have the opposite effect. 18 The production of ROS is a crucial mechanism implicated in the activation of the NLRP3 inflammasome. 19-20 Furthermore, adding antioxidants significantly reduces the activation of the NLRP3 inflammasome and cell pyroptosis. 21

Hydrogen (H_2_) is an important biological regulator that has antioxidant properties. 22-23 The hydrogen molecule is small, electrically neutral, and nonpolar. These properties allow it to diffuse through cell membranes and lipid bilayers and reach the subcellular compartments, such as mitochondria, which is the primary site of ROS generation. Our previous research shows that H_2_ attenuates doxorubicin-induced myocardial injury and I/R injury by alleviating inflammation and oxidative stress. [24-25]Many studies have also shown that H_2_ treatment could reduce infarct size in I/R injury and isoproterenol (ISO)-induced myocardial infarction. 26-28 Furthermore, a single-center prospective, open-label, blinded study has demonstrated that inhalation of H_2_ during percutaneous coronary intervention results in left ventricular reverse remodeling at six months after ST-elevated myocardial infarction. 29 However, the protective effect of H_2_ on AMI and its specific mechanism remain poorly defined.

This study aimed to estimate the protective effects of H_2_ on myocardial infarction in rats and association with the amelioration of oxidative stress and NLRP3 inflammasome mediated pyroptosis.

## Material and methods

### Animals

All procedures were maintained in accordance with the guidelines of the NIH (Guide for the Care and Use of Laboratory Animals) and approved by the Animal Ethics Committee of the Fourth Affiliated Hospital of Harbin Medical University. Sixty-four male Wistar albino rats (weight, 180-200 g) were obtained from the Laboratory Animal Center of the Second Affiliated Hospital of Harbin Medical University and raised in the First Affiliated Hospital of Harbin Medical University Animal Center in a temperature- and light-controlled environment (20-25°C and 12-12h light-dark cycle).

### Experimental Protocol

Studies have reported that 2% H_2_ is safe and therapeutic.^30^-^31^ Additionally, there is an explosion risk with high levels of hydrogen; therefore, 2% H_2_ was selected for this study.

Sixty-four rats were randomly divided into three groups: sham-operated (Sham, n=20), acute myocardial infarction (AMI, n=22), and hydrogen (H_2_, n=22). All rats fasted for 12 h before the start of the experiment. The AMI rat model was established *via* left anterior descending coronary artery (LAD) ligation with the rats under general anesthesia induced by 1% pentobarbital sodium (40 mg/kg). Tracheal intubation was performed after cleaning the respiratory secretions and connected with the small animal breathing-anesthesia machine. A left lateral thoracotomy was performed, and the pericardium was opened to expose the LAD. The LAD was ligated 2-3 mm from the tip of the left auricle with a 6-0 nylon suture. The ligation width was approximately 3 mm. Coronary occlusion was confirmed with an elevated ST-segment change on the electrocardiogram. The Sham group was threaded without ligation, while the LAD was ligated in the AMI and H_2_ groups. In addition, 2% H_2_, which was prepared and estimated as previously described 32, was administered by inhalation for 24 h after the ligation of the left coronary artery in the H_2_ group.

### Echocardiography

All animals were anesthetized after 24 h and fixed in the supine position for transthoracic echocardiography detection. The interventricular septal thickness at diastole (IVSd), left ventricular internal diameter in diastole and systole (LVIDd and LVIDs), left ventricular posterior wall at diastole (LVPWd), ejection fraction (EF) and shortening fraction (FS) were evaluated by an animal-specific Doppler ultrasound system (S12-4, Philips CX50, Holland). All measurements are averaged from five consecutive cardiac cycles.

### Measurement of Serum parameters

After the transthoracic echocardiography detection, the blood samples were immediately collected from rats' aorta, centrifuged at 3000rpm for a period of 15 min at 4°C, and then were stored at -80°C to determine the serum parameters. The levels of cardiac troponin I (TnI) (Nanjing Jiancheng BioEngineering Institute, China), brain natriuretic peptide (BNP) (Nanjing Jiancheng BioEngineering Institute, China), and ROS (Cusabio Biotech. Co. Ltd, China) were detected by enzyme-linked immunosorbent assay (ELISA), according to the manufacturers' instructions.

### Malondialdehyde (MDA) Concentration in Cardiac Tissue

After blood sample collection, left ventricular tissue below the ligature of rats was immediately harvested and washed in cold PBS. The concentration of cardiac MDA was detected using a commercial kit, as per the manufacturer's instructions (KeyGEN Biotech. Co. Ltd, China).

### Hematoxylin and eosin (H&E) staining

After blood sample collection, animals were sacrificed and the left ventricular tissue below ligature was immediately harvested. The collected heart tissue was washed in cold phosphate buffer saline (PBS) and a 2 mm thick slice was fixed in 4% formalin, embedded in paraffin, and serially sectioned at 2μm for routine H&E staining (Beyotime, China). Five random sections per heart were assessed, using 5 separate fields in each under a light microscope (DP73, Olympus Co, Japan) by 3 pathologists who were blinded to the grouping of the rats.

### Triphenyltetrazolium chloride (TTC) staining

TTC staining was utilized to evaluate the infarct area. The collected hearts were rapidly washed in cold saline. Next, the heart tissues below the ligature were sliced into five slices and then rapidly stained with 2% TTC (Solarbio, China) according to the manufacturers' instructions. The images were pictured by digital camera and analyzed using Image J software. The percent of cardiac infarct area was calculated as the white area/total area ×100%.

### Immunohistochemical Examinations

Immunohistochemical staining of the cardiac tissue was performed using specific antibodies for the expression of 8-hydroxy-2-deoxyguanosine (8-OHdG). A 2 mm thick slice of cardiac tissue was sectioned, fixed in 4% Paraformaldehyde, and paraffin embedded. The sections were heated at 100 ºC for antigen retrieval with 0.1 M sodium citrate and blocked with hydrogen peroxide. After washing with PBS, the sections were incubated with anti-8-OHdG polyclonal antibody (1:200 dilution, bs-1278R, Bioss) at 4 ºC, followed by conjugation to the secondary antibody(SP-0023, Bioss) and 3, 3-diaminobenzidine (DAB) staining. Finally, sections were detected and analyzed using a light microscope (DP73, Olympus Co, Japan).

### Terminal Deoxynucleotidyl Transferase-Mediated dUTP-Biotin Nick End Labeling (TUNEL) Assay

TUNEL assay was performed on cardiac tissue sections according to the manufacturer's instructions (Roche, Switzerland). The sections were observed under a light microscope (DP73, Olympus Co., Japan) and TUNEL-positive cells were counted using Image J software.

### Western Blot Analysis

Western blot was performed as described previously. 24 Specific proteins were detected using the following primary antibodies: NLRP3 (no. ab214185, 1:1000, Abcam), ASC (no. A1170, 1:1000, ABclonal), caspase-1 (no. ab238972, 1:1000, Abcam), IL-1β (no. ab9722, 1:1000, Abcam) and GSDMD (no. ab219800, 1:1000, Abcam). Horseradish peroxidase-conjugated secondary antibodies (ZB-2301, ZB-2305, 1:1000, ZSGB) were incubated with the membrane and the antibody complexes were detected by Imaging System (Bio-Rad, Hercules, CA, USA). β-Actin (no.TA-09, 1:1000, ZSGB) was used as the control to detect equal protein loading.

### Statistical Analysis

The results are presented as mean ± standard deviation (SD). One-way analysis of variance (ANOVA) was used for multiple comparisons, with a post-hoc. Student-Newman-Keuls tests (GraphPad Prism 6.0). An unpaired *t*-test was used to compare two groups. *P* <0.05 was considered statistically significant.

## Results

### Hydrogen Treatment Ameliorates AMI-induced Myocardial Injury

**Figure 1:**
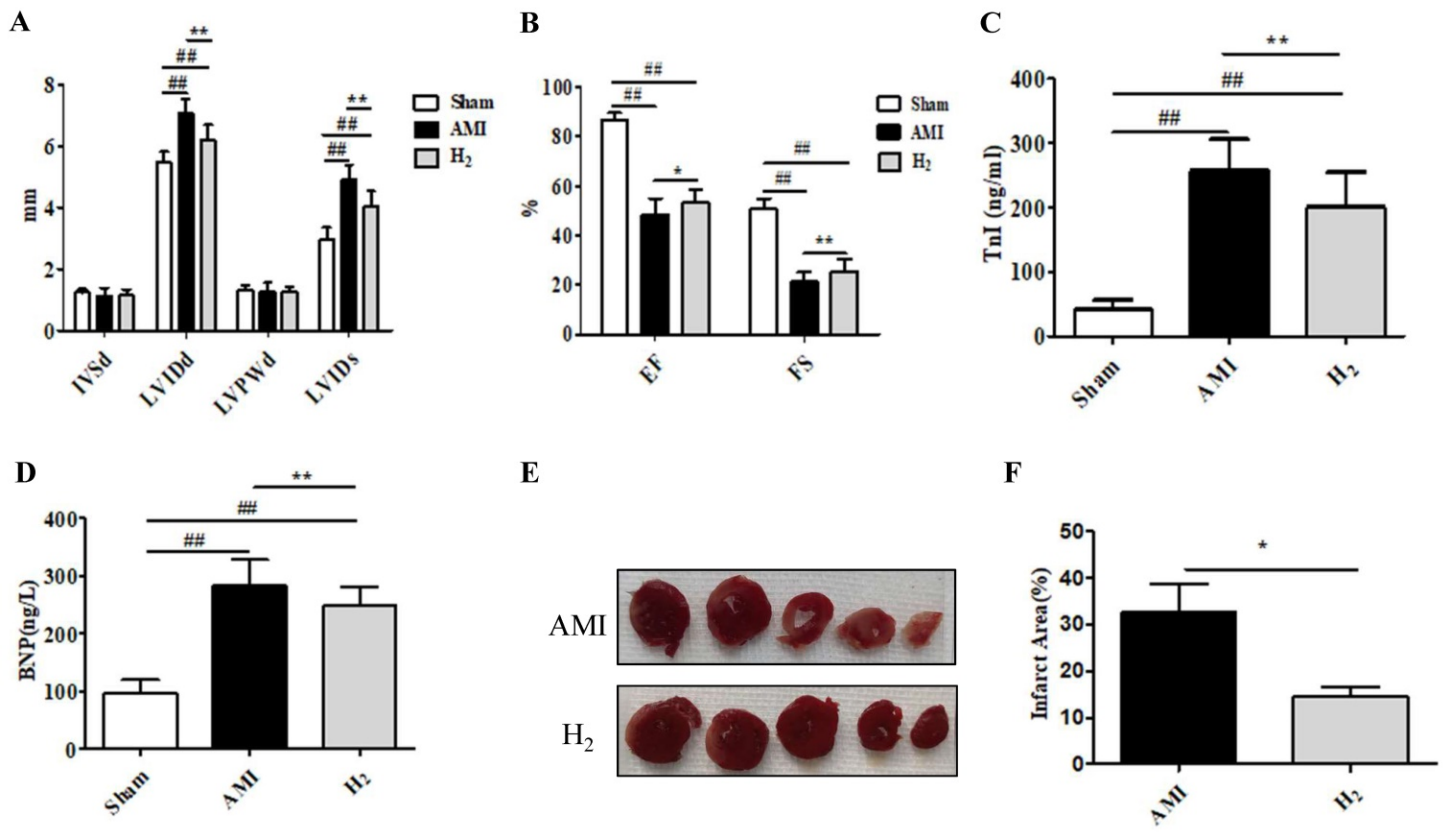
**Hydrogen ameliorates AMI-induced cardiac injury.** (A) IVSd, LVIDd, LVPWd, and LVIDs in each group (n =20). (B) EF and FS in each group (n =20). (C) Serum TnI in each group (n =20). (D) Serum BNP in each group (n =20). (E) TTC cardiac tissue stainings. (F) The percentage infract area characterized with TTC staining (n =5). ## *P* < 0.01 *versus* Sham group; * *P* < 0.05 *versus* AMI group; **P < 0.01 *versus* AMI group.

To estimate the cardioprotective effect of hydrogen in AMI rats, cardiac function was detected by echocardiography. Compared with the Sham group, the LVIDd and LVIDs was significantly increased in the AMI group (*P* < 0.01; Figure 1A), whereas both decreased markedly after hydrogen inhalation (*P* < 0.05); however, the differences were not significant between the three groups (*P* > 0.05). The EF and FS were significantly reduced in the AMI group (*P*<0.01; Figure 1B); however, hydrogen treatment increased the EF (*P* < 0.05) and FS (*P* < 0.01) when compared in the AMI group (Figure 1B). Furthermore, there was an increase in serum TnI and BNP concentration in the AMI group when compared with the Sham group(*P* < 0.01; Figure 1C,1D). By contrast, serum TnI (*P* < 0.05; Figure 1C) and BNP (*P* < 0.01; Figure 1D) concentrations were decreased in the H_2_ group. In addition, TTC staining of heart tissue revealed that a smaller AMI-induced infarct area in the H_2_ group when compared with the AMI group (*P* < 0.05; Figures 1E,1F). These results indicated that hydrogen treatment could effectively relieve AMI-induced myocardial injury and cardiac dysfunction.

### Hydrogen Treatment Inhibits AMI-Induced Cardiac Oxidative Stress

**Figure 2:**
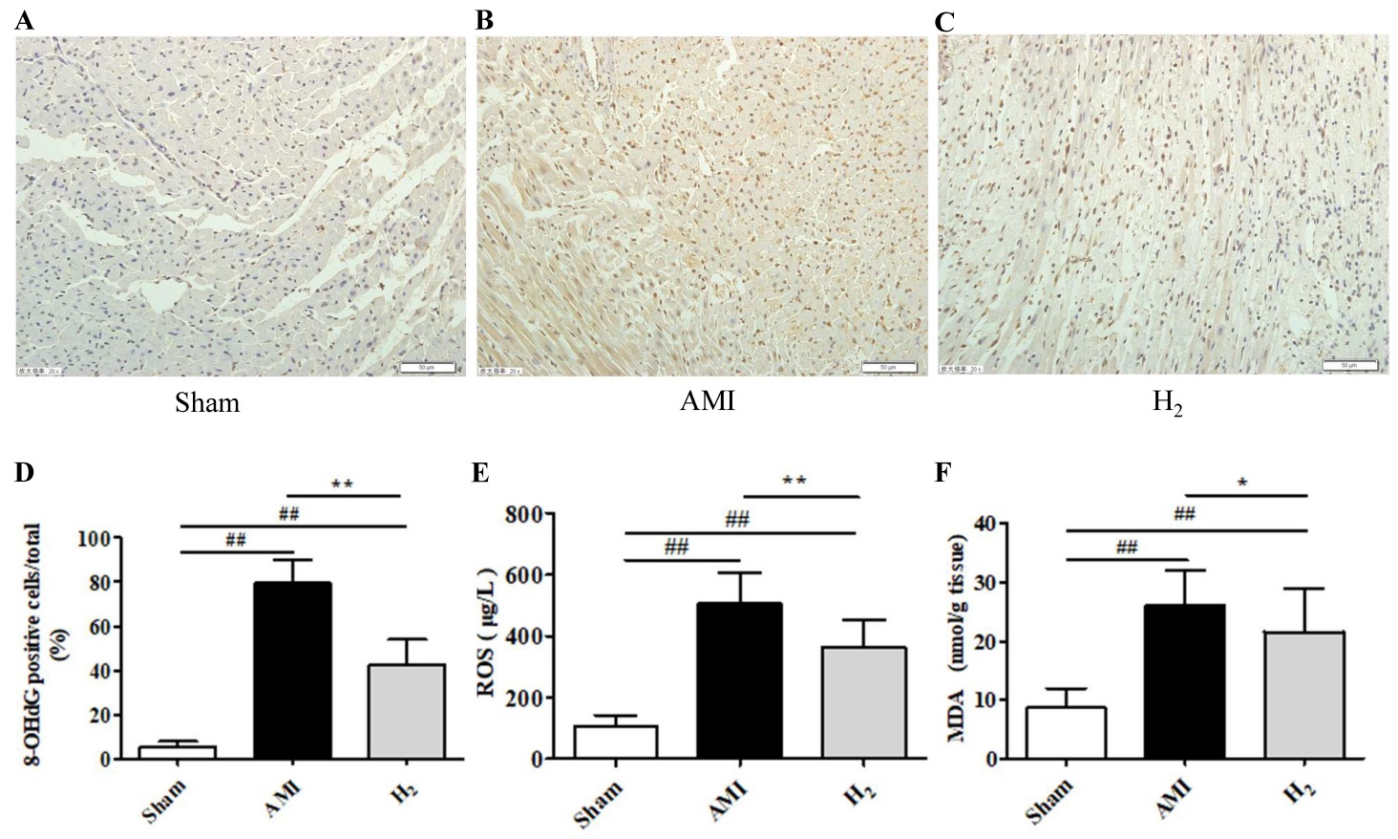
** Oxidative stress markers of cardiac tissue in each group.** (A-C) Cardiac 8-OHdG staining in each group (200×). (D) The percentage of 8-OHdG-positive cells (brown staining) in heart tissue (n = 3). (E) Serum ROS levels in each group (n = 20). (F) Cardiac MDA levels in each group (n = 14). ## *P* < 0.01 *versus* Sham group; * *P* < 0.05 *versus* AMI group; **P < 0.01 *versus* AMI group.

To understand the effects of hydrogen on oxidative processes during AMI, the products of oxidative stress (8-OHdG, ROS, and MDA) were detected in the cardiac tissue and serum. The expression of 8-OHdG, ROS, and MDA were markedly higher in the AMI group than in the Sham group (*P* < 0.01; Figure 2). However, compared with the AMI group, cardiac 8-OHdG (*P* < 0.01; Figure 2A-D), ROS (*P* < 0.01; Figure 2E) and MDA (*P* < 0.05; Figure 2F) were significantly reduced in the H_2_ group.These results suggested that hydrogen treatment could inhibit AMI-induced cardiac oxidative stress.

### Hydrogen Treatment Ameliorates the Cardiac Inflammatory Response and Cardiomyocytes DNA Damage Induced by AMI

**Figure 3:**
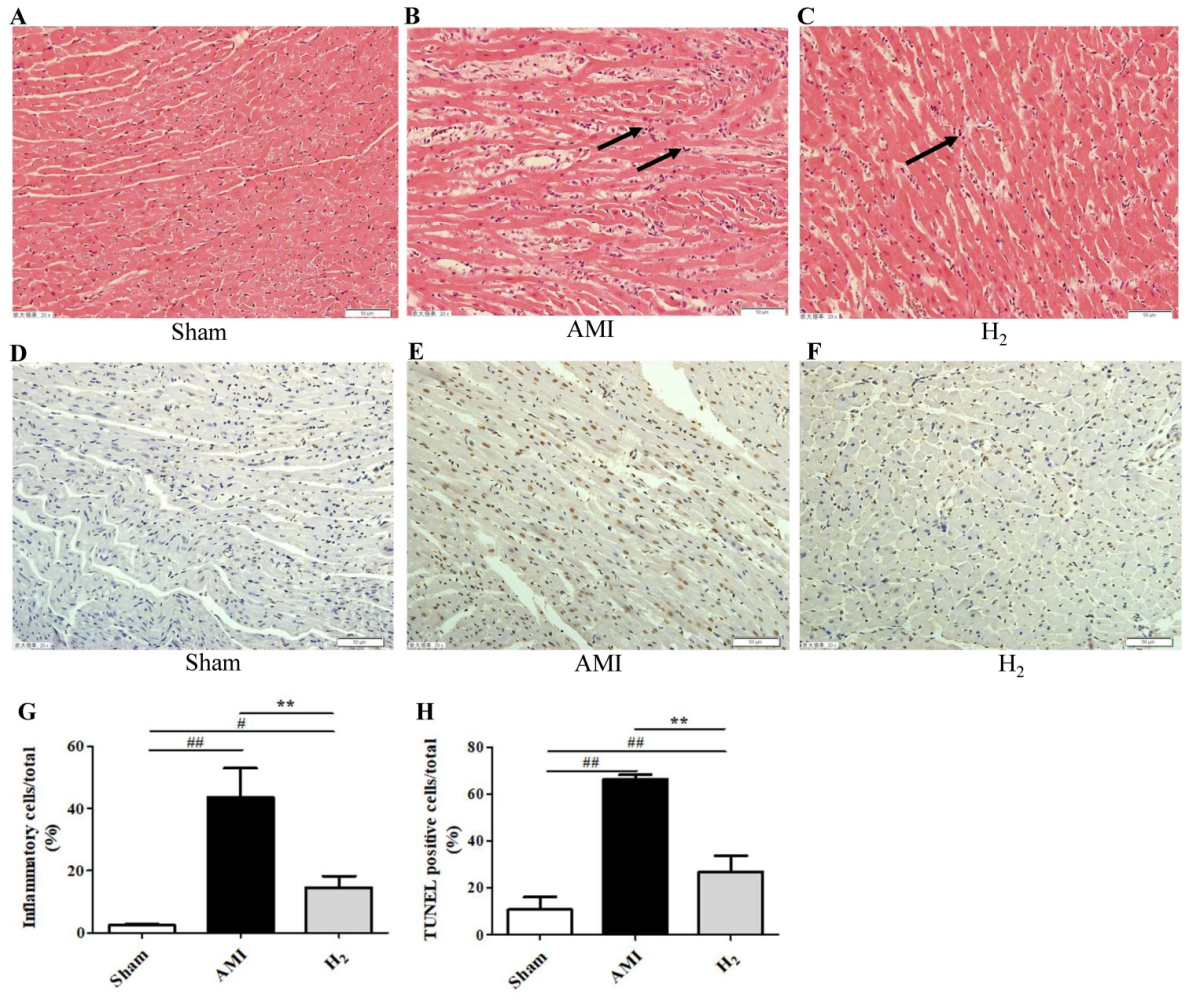
** Cardiac H&E staining and TUNEL staining in each group.** (A-C) H&E staining of cardiac tissue in each group. (D-F) Cardiac TUNEL staining of each group (200×, long black arrows for inflammatory infiltration). (G-H) The percentage of inflammatory cells or TUNEL-positive cells (brown staining) of heart tissue ( n = 3, # *P* < 0.05 *versus* Sham group; ## *P* < 0.01 *versus* Sham group; ** *P* < 0.01 *versus* AMI group).

H&E staining revealed obvious infiltration of inflammatory cells in AMI group compared with that in Sham group (*P* < 0.01; Figures 3A, 3B, 3G), while it was ameliorated in the H_2_ group (*P* < 0.01; Figures 3C, 3G). Those results indicated that hydrogen could attenuate AMI induced cardiac inflammation. Furthermore, there were significantly more TUNEL-positive cells in the cardiac tissue of AMI rats when compared with the Sham group (*P* < 0.01; Figures 3D, 3E, 3H), which was attenuated after hydrogen treatment (*P* < 0.01; Figures 3E, 3F, 3H). This indicated that hydrogen treatment significantly reduced AMI-induced DNA damage in cardiomyocytes.

### Hydrogen Treatment Regulates AMI-Induced NLRP3 Inflammasome Activation

**Figure 4:**
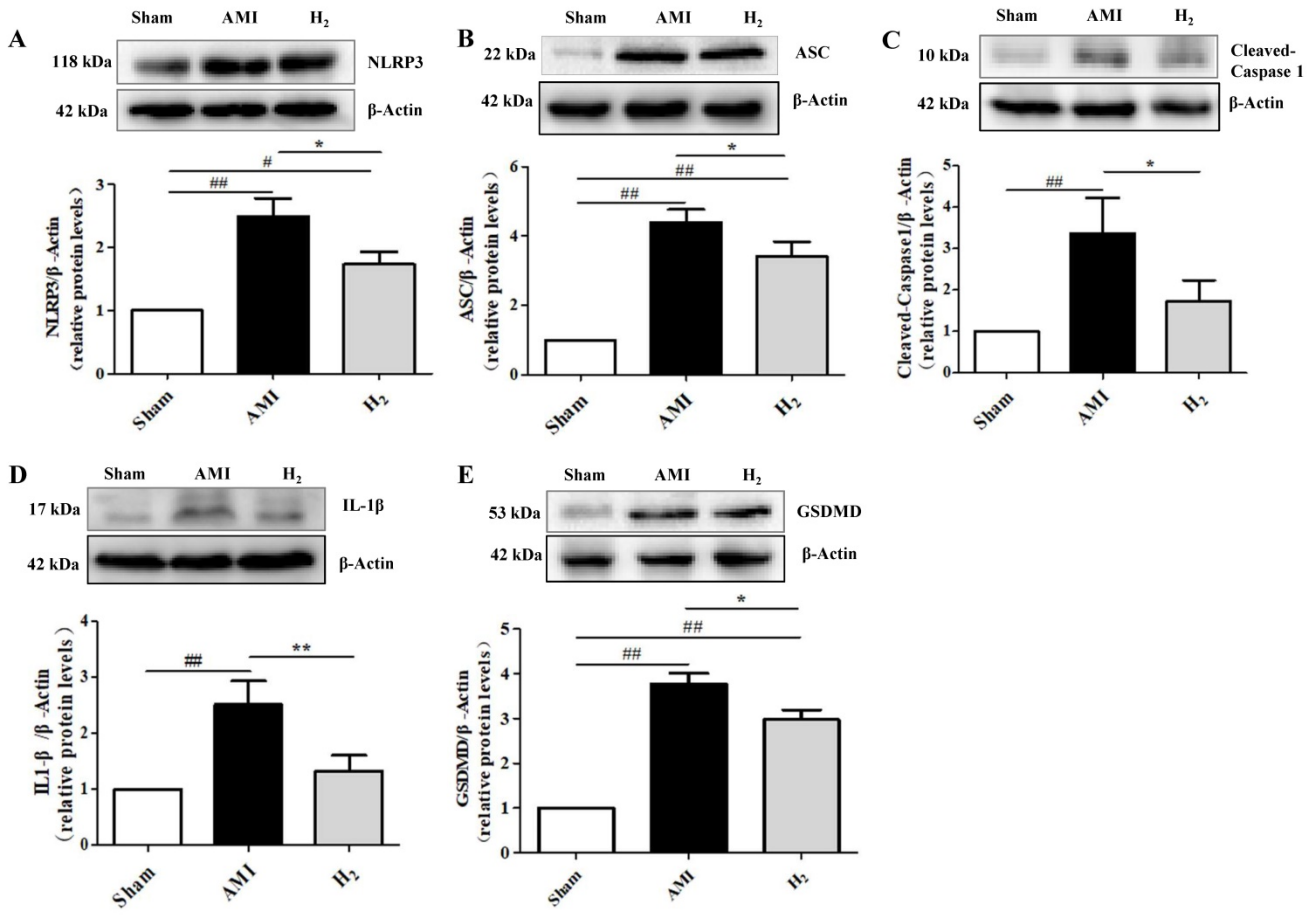
** Pyroptosis-related protein expression in cardiac tissue.** (A) Cardiac NLRP3 expression in each group (n = 3). (B) Cardiac ASC expression in each group (n = 3). (C) Cardiac caspase-1 expression in each group (n = 3). (D) Cardiac IL-1β expression in each group (n = 5). (E) Cardiac GSDMD expression in each group (n = 3). ## *P* < 0.01 *versus* Sham group; # *P* < 0.05 *versus* Sham group; * *P* < 0.05 *versus* AMI group; ** *P* < 0.01 *versus* AMI group.

Studies showed that hypoxia induces the activation of NLRP3 inflammasomes, which is related to ROS production.^33^ Furthermore, NLRP3 inflammasome-dependent inflammation promotes harmful left ventricular remodeling after AMI. 34 To investigate the effect of hydrogen on NLRP3 inflammasome activation in AMI rats, the expression of NLRP3 and its related proteins in cardiac tissue were detected by western blot. We found that of NLRP3 expression was markedly higher in the AMI group when compared with the Sham group (*P* < 0.01; Figure 4A). By contrast, it was significantly reduced in the H_2_ group when compared with the AMI group (*P* < 0.05; Figure 4A). In addition, the expression of ASC (Figure 4B), caspase-1 (Figure 4C), IL-1β (Figure 4D) and GSDMD (Figure 4E) in AMI group were considerably increased when compared with the Sham group (*P* < 0.01), but they were all decreased by hydrogen treatment. These findings indicate that hydrogen could inhibit the AMI induced NLRP3 inflammasome expression and cardiomyocyte pyroptosis.

## Discussion

Myocardial infarction remains a leading cause of death worldwide and current treatment can only delay the disease progression. Hydrogen, when used as a therapeutic gas, is cardioprotective. In this study, we used a rat model of AMI to investigate the effects and mechanisms of hydrogen protection in the treatment of AMI. Our major findings were as follows: (1) Hydrogen improved the cardiac functions and reduced infarct size in rats after AMI; (2) the therapeutic effects of hydrogen were associated with ameliorating oxidative stress and NLRP3 inflammasome-mediated pyroptosis.

Acute high dose ISO can cause severe myocardial stress and induce infarct-like necrosis. Sun *et al*. have found that hydrogen relieves oxidative stress, reduce serum inflammatory cytokines levels, and infarct size via single intraperitoneal injection of hydrogen rich saline in ISO-induced myocardial infarction rats. 28 Here, we applied acute interruption of the blood supply caused via coronary artery ligation because it was more consistent with the pathological process of AMI. Furthermore, when continuous hydrogen inhalation was administered to increase the duration of hydrogen in* vivo*, similar cardiac protective effects were observed.

Inflammation plays a crucial role in the pathogenesis of acute coronary events. 35 Inflammation in myocardial infarction occurs in the absence of infectious agents and is therefore known as “sterile inflammation”. 36 This is mediated through the NLRP3 inflammasome. Inhibition of this inflammasome reduces infarct size and preserves cardiac function in a pig model of I/R injury.^37^ The generation of ROS is one of the upstream pathways of NLRP3 inflammasome activation. 21 In view of the antioxidant properties of hydrogen, we hypothesize that it can reduce the oxidative stress caused by myocardial ischemia in rats. This reduces ROS-mediated activation of NLRP3 inflammasome and subsequent production of IL-1β, thereby resulting in the alleviation of inflammatory response in the myocardium. Ultimately, the infarct size is decreased, and cardiac function is improved.

Previous studies assessing the effect of hydrogen on myocardial infarction have focused primarily on I/R injury because of the importance of oxidative stress in I/R injury. However, limited medical resources or severe coronary artery lesion meant that some AMI patients cannot receive timely reperfusion treatment. In addition, ischemic regions of the myocardium remain in nearly two-thirds of patients even after successful recanalization due to MVO. Therefore, it is important to find a new strategy for the treatment of AMI. This study revealed the protective effect of hydrogen on AMI, which may be related to the inhibition of oxidative stress and alleviation of NLRP3 inflammasome-mediated pyroptosis. Taken together, these results offer a new effective intervention for the treatment of AMI.

There are some limitations to the study that should be noted. First, although we demonstrated that hydrogen could reduce activation of the NLRP3 inflammasome and improve cardiac function and remodeling after AMI, it is unknown whether NLRP3 plays a causal role. Therefore, investigating the effect of hydrogen on AMI in NLRP3-deficient rat would further verify whether the protective effect of hydrogen is limited to the inhibition of NLRP3. Second, the present study used male rats and there is no clarity about whether the same results can be obtained using female rats. Third, there are various patterns of cell death in myocardial infarction, including necrosis, apoptosis, necroptosis, autophagy-related cell death, pyroptosis, and ferroptosis. 38 It remains unclear which cell death programs are most important and we did not make any further evaluation of cell death patterns beyond pyroptosis in the present study.

## Conclusion

Our present study estimates the cardioprotective effect of hydrogen on an AMI rat models. We demonstrated that hydrogen might reduce NLRP3-mediated cardiomyocytes pyroptosis by inhibiting oxidative stress, thereby reducing myocardial injury and improving cardiac function. Owing to its safety and efficacy, hydrogen inhalation may be considered as a potential therapy for AMI induced myocardial injury and applied to patients with AMI.

### Funding

This work was supported in part by Postdoctoral Fund of Heilongjiang (Project No. LBH-Z18191) and National Natural Science Foundation of China (Grant No. 81871491).
